# Comparative enzymatic hydrolysis of porcine tracheal cartilage generates ACE/DPP-IV-inhibitory and antioxidant peptides with enhanced bioactivity following simulated gastrointestinal digestion and molecular docking analysis^☆^

**DOI:** 10.1016/j.fochx.2026.104298

**Published:** 2026-08-09

**Authors:** Rayudika Aprilia Patindra Purba, Suchanya Sinsranoi, Phanthipha Laosam, Phornpilat Senanok, Pichitpon Luasiri, Pornphutthachat Sota, Nattapol Pongsamai, Saranya Suwanangul, Jukkrapong Pinyo, Sasikan Katemala, Saruttiwong Boonkong, Kamonpan Sanachai, Papungkorn Sangsawad

**Affiliations:** a Department of Agricultural Science and Technology, Faculty of Innovative Agriculture, Fisheries and Food, Prince of Songkla University, Surat Thani Campus, Surat Thani, 84000, Thailand; b Research and Development Institute Suranaree University of Technology, Nakhon Ratchasima 30000, Thailand; c Postharvest Technology and Innovation in Animal Unit, Institute of Agricultural Technology, Suranaree University of Technology, Nakhon Ratchasima 30000, Thailand; d School of Animal Technology and Innovation, Institute of Agricultural Technology, Suranaree University of Technology, Nakhon Ratchasima 30000, Thailand; e Suranaree University of Technology Hospital, Nakhon Ratchasima 30000, Thailand; f Program in Food Science and Technology, Faculty of Engineering and Agro-industry, Maejo University, Chiang Mai 50290, Thailand; g Faculty of Agriculture at Kamphaeng Saen, Kasetsart University, Kamphaeng Saen Campus, Nakhon Pathom 73140, Thailand; h Program in Animal Science, Faculty of Science and Technology, Loei Rajabhat University, Loei 42000, Thailand; i Department of Biochemistry, Faculty of Science, Khon Kaen University, Khon Kaen 40002, Thailand

**Keywords:** Bioactive peptides, Porcine tracheal cartilage, Enzymatic hydrolysis, Angiotensin-converting enzyme inhibition, Dipeptidyl peptidase-IV inhibition, Antioxidant activity, In vitro gastrointestinal digestion

## Abstract

Porcine tracheal cartilage, an underutilized Type II collagen-rich by-product, was systematically evaluated as a source of angiotensin I-converting enzyme (ACE) inhibitory, dipeptidyl peptidase-IV (DPP-IV) inhibitory, and antioxidant peptides. Neutrase at 4 h outperformed Papain in generating ACE-inhibitory and antioxidant peptides. The <3 kDa ultrafiltration fraction, enriched in small hydrophobic peptides, exhibited the highest bioactivities. LC-MS/MS identified eight sequences (870–1565 Da). Chemical synthesis confirmed LGLGADMFHR as the most potent ACE inhibitor (IC_50_ = 0.52 mM), QGLPGPGAVAEYT as the lead DPP-IV inhibitor (IC_50_ = 1.84 mM), and LGFGADRAGPQ as the strongest antioxidant (5.64 mM Trolox equivalents). Molecular docking revealed LGLGADMFHR binding through Zn^2+^ coordination and hydrogen bonds with Cys352, Glu376, and Asp453. Standardised INFOGEST gastrointestinal digestion progressively enhanced ACE inhibition and antioxidant activities while DPP-IV inhibition declined, establishing porcine tracheal cartilage as a viable source of gastrointestinally stable cardiovascular bioactive peptides.

## Graphical abstract

**Figure:**
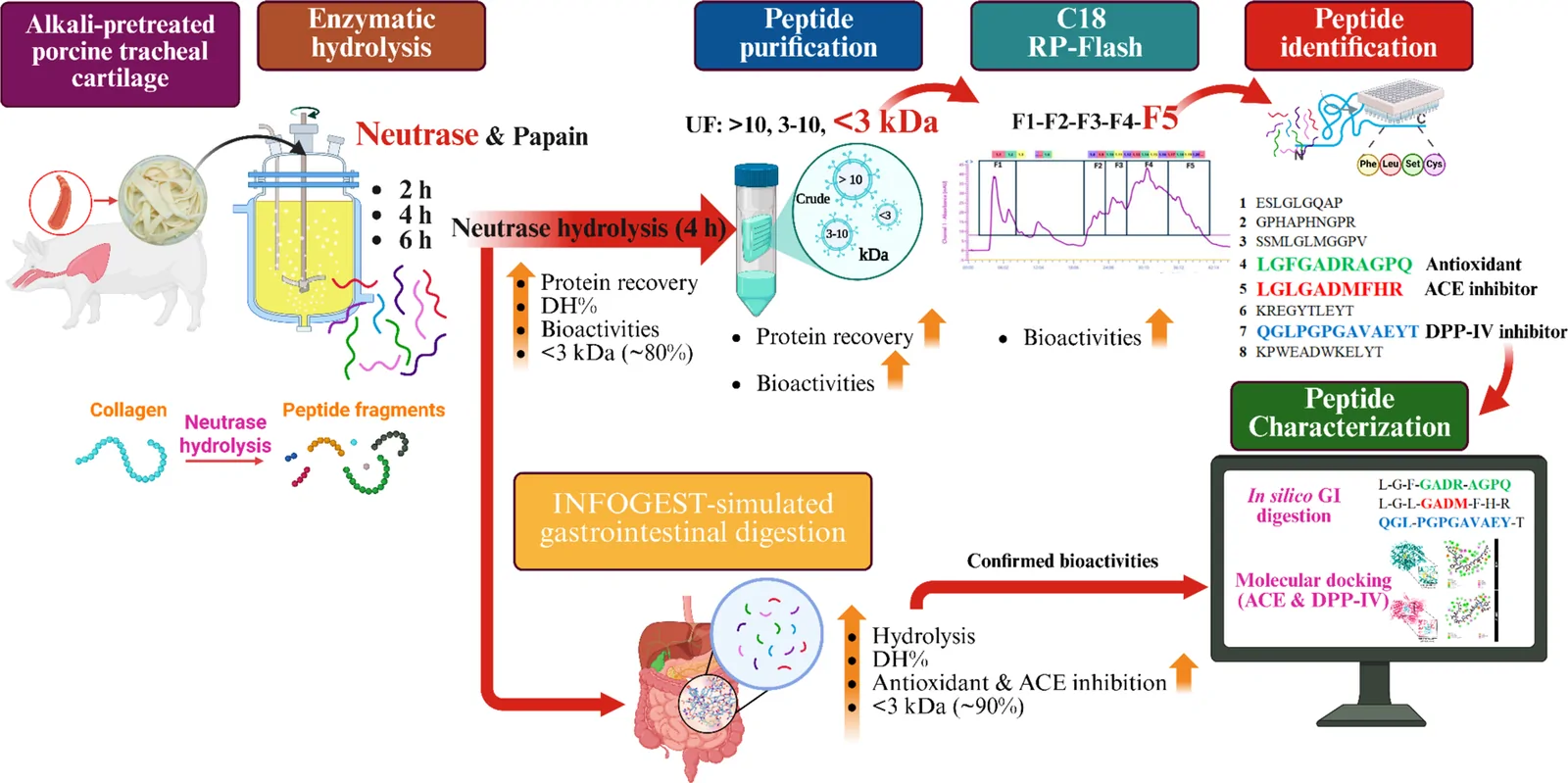

## 1 Introduction

The global meat processing industry generates approximately 50 million metric tons of by-products annually, representing 30–40% of livestock carcass weight (Cedeno et al., 2025). Enzymatic hydrolysis transforms these protein-rich materials into bioactive peptides with health-promoting properties, including angiotensin I-converting enzyme (ACE) inhibition for blood pressure regulation, dipeptidyl peptidase-IV (DPP-IV) inhibition for glycaemic control, and antioxidant capacity (Purba et al., 2025; Udenigwe & Aluko, 2012). Because the synthetic drugs conventionally used for blood pressure and glycaemic control can cause adverse effects, food-derived peptides offer a safer natural alternative and a compelling direction for functional ingredient development (Chen et al., 2025; Musaimi, 2024).

Collagen-rich tissues constitute a major fraction of slaughterhouse by-products, yet skin, bone, and fish sources have been studied far more than tracheal cartilage (Singh et al., 2025). Tracheal cartilage is predominantly an extracellular matrix in which Type II collagen constitutes approximately 90–95% of the total collagen fraction, compositionally distinct from the Type I collagen of most studied sources (X. Hu et al., 2024); this divergence, together with its markedly lower fat content, may yield novel peptide sequences upon enzymatic cleavage. Research on porcine tracheal cartilage has so far centred on its structural and nutraceutical components as its biochemical composition and mechanical properties have been characterized (Hoffman et al., 2016), and it serves as a raw material for Type II collagen and chondroitin sulfate, including a commercially hydrolysed ingredient used for joint health. These applications, however, exploit the intact collagen and glycosaminoglycan fractions. The enzymatic hydrolysate of porcine tracheal cartilage has not been evaluated for ACE-inhibitory, DPP-IV-inhibitory, or antioxidant peptides, and its constituent peptide sequences remain unidentified. Because collagen-derived peptides are generally well absorbed, owing to their proline- and hydroxyproline-rich di- and tripeptides, and porcine skin collagen hydrolysate shows total hydroxyproline absorption comparable to, and modestly higher than, that of fish collagen in humans (Virgilio et al., 2024), similarly favourable oral bioavailability can be anticipated for the proline-rich peptides of tracheal cartilage. Coupled with the status of trachea as one of the most underutilized animal by-products (Kim et al., 2025; Seob et al., 2025), this represents a clear and unaddressed gap that the present study targets.

Papain and Neutrase were selected because both are active at the natural pH of the tracheal cartilage slurry (6.5–6.8), allowing hydrolysis to proceed without acid or base addition and thereby avoiding the excess salt (NaCl) formation and cost-intensive desalination that would otherwise burden downstream food applications. Papain, a cysteine protease from *Carica papaya*, exhibits broad substrate specificity with a preference for hydrophobic amino acids and has been widely employed to generate ACE-inhibitory, DPP-IV-inhibitory, and antioxidant peptides from collagen-rich substrates, including porcine skin, bovine bone, and fish by-products (Hong et al., 2019). Neutrase, a neutral metalloprotease from *Bacillus subtilis*, has a near-neutral pH optimum and distinct cleavage patterns, and has likewise been reported to liberate potent ACE-inhibitory and antioxidant peptides from animal-derived proteins, often outperforming other commercial proteases under mild processing conditions (Damrongsakkul et al., 2008). The two enzymes thus belong to mechanistically distinct classes while both acting on the same substrate at its natural pH, enabling a direct comparison of how enzyme class shapes the peptide populations and bioactivities generated. Alcalase, although widely used for bioactive-peptide production, was deliberately excluded because its alkaline pH optimum (typically operated at pH 8.0) would require alkalising and subsequently neutralising the substrate, generating excess NaCl and necessitating a desalination step that conflicts with the salt-free strategy adopted here. Other proteases requiring markedly acidic or alkaline conditions were omitted on the same basis. Recent comparative studies on duck blood (Laosam et al., 2024) and porcine blood (Moreno-Mariscal et al., 2025) further show that different proteases yield divergent bioactivity outcomes, challenging the assumption that a higher degree of hydrolysis universally predicts superior functionality. Such systematic enzyme comparisons nonetheless remain scarce, even though enzyme choice governs the identity and activity of the resulting peptides, not merely hydrolysis efficiency.

Molecular weight is a further determinant of peptide bioactivity. This includes small peptides (typically <3 kDa) that often show enhanced ACE inhibition owing to better access to the enzyme active site (W. Hu et al., 2025), yet size–activity relationships have not been characterized for tracheal cartilage hydrolysates. Gastrointestinal stability is equally important and frequently overlooked, since many peptides that are active in vitro are degraded by gastric and intestinal proteases (Zhou et al., 2025). Although standardised protocols such as INFOGEST 2.0 (Brodkorb et al., 2019) have improved reproducibility, comprehensive studies tracking molecular weight transformations and multiple bioactivities throughout complete gastrointestinal digestion remain scarce for most protein sources, and for tracheal cartilage hydrolysates no such data exist.

This investigation therefore systematically evaluated porcine tracheal cartilage as a substrate for bioactive peptide generation, integrating comparative protease evaluation, size-dependent fractionation, and peptide characterization to establish structure–activity relationships governing ACE inhibition, DPP-IV inhibition, and antioxidant capacity. Simulated gastrointestinal digestion then assessed whether the resulting peptides retain functionality during digestive proteolysis, a prerequisite for oral bioavailability.

## 2 Materials and methods

### 2.1 Materials and reagents

Neutrase (0.8 Anson units per gram) and Papain enzymes (≥6000 USP units per mg protein) were procured from Brenntag (Bangkok, Thailand) and served as proteolytic enzymes for hydrolysis. For simulated gastrointestinal digestion, porcine pepsin (≥2500 U per mg protein, catalogue number P7000) and pancreatin (8× USP specifications, catalogue number P7545) were obtained from Sigma-Aldrich (St. Louis, MO, USA). Dipeptidyl peptidase IV (DPP-IV; ≥10 U per mg protein, from porcine kidney, catalogue number D7052), Gly-Pro p-nitroanilide hydrochloride, angiotensin I-converting enzyme (ACE) from human (≥2.0 U per mg protein, catalogue number SAE0075), N-[3-(2-furyl)acryloyl]-Phe-Gly-Gly (FAPGG), acetonitrile, trifluoroacetic acid, picrylsulfonic acid (TNBS), cytochrome C (12,000 Da), and maltodextrin were obtained from Chemipan Corporation Co., Ltd. (Bangkok, Thailand). Porcine tracheal cartilage was purchased from Moocharoen Food Service Co., Ltd. (Nakhon Ratchasima, Thailand) and stored at −20 °C until analysis.

### 2.2 Preparation of alkaline-pretreated porcine tracheal cartilage

Minced porcine tracheal cartilage (2 kg) was subjected to alkaline pretreatment with 0.1 M NaOH at a 1:10 ratio (*w*/*v*) at room temperature (∼25 °C) for 2 h, to remove non-collagenous proteins and surface lipids and thereby enrich the collagen-rich protein fraction prior to hydrolysis. The treatment was repeated twice until no visible surface lipid remained (Liu et al., 2015). The slurry was filtered through muslin cloth and washed extensively with deionized water until reaching neutral pH (7.0 ± 0.2). Gravimetric measurement of the final washed and freeze-dried solids indicated a cumulative solid loss of approximately 21.5% (dry-weight basis) over the complete pretreatment, that is, the alkaline treatment together with the subsequent water washing. Throughout processing, samples were maintained at −20 °C. Proximate composition (moisture, protein, fat, and ash) of both the untreated cartilage and the alkaline-pretreated substrate was determined following AOAC (2016) methods, with fat content specifically determined by the method of Folch et al. (1957) using chloroform:methanol (2:1, *v*/v) as the extraction solvent. Protein content was calculated from Kjeldahl nitrogen using a nitrogen-to-protein conversion factor of 5.5, appropriate for the collagen-rich composition of cartilage.

### 2.3 Enzymatic hydrolysis

To conduct enzyme screening (Papain vs. Neutrase), alkaline-pretreated minced porcine tracheal cartilage (50 g, wet weight; corresponding to 8.33 g dry solids (8.09 g protein) based on the composition in Table 1) was mixed with deionized water (25 mL) and heated to 90 °C for 30 min under continuous stirring to thermally denature the collagen. After cooling to the optimal temperature for each enzyme (50 °C for Neutrase; 65 °C for Papain), enzymes were added at 5% (enzyme/substrate, *w*/w). The hydrolysis was performed without external pH adjustment. The natural pH of the tracheal cartilage slurry (6.5–6.8) falls within the functional activity ranges for both enzymes (Neutrase optimal pH 6.0–8.0, Papain optimal pH 5.0–7.0). This strategy specifically avoided the addition of acids or bases to prevent the formation of excess salt (NaCl), which would otherwise impair the sensory quality (excessive saltiness) and necessitate cost-intensive desalination steps. Hydrolysis was conducted for 2, 4, and 6 h under constant agitation. Reactions were terminated by heating to 90 °C for 30 min to inactivate enzymes. Hydrolysates were centrifuged at 5000 ×*g* for 10 min at 4 °C, and supernatants were collected for analysis. Samples were stored at −20 °C until further use.

**Table 1:** Proximate composition of porcine tracheal cartilage before (untreated) and after alkaline pretreatment.

Proximate composition (%)	Untreated – wet basis	Untreated – dry basis	Alkaline-pretreated – wet basis	Alkaline-pretreated – dry basis
Moisture	81.96 ± 0.57 ^a^	–	83.34 ± 0.93 ^a^	–
Protein	14.31 ± 0.70 ^b^	76.24 ± 2.16 ^a^	17.97 ± 0.76 ^b^	97.11 ± 0.32 ^a^
Fat	3.67 ± 0.76 ^c^	19.40 ± 2.56 ^b^	0.21 ± 0.03 ^d^	1.15 ± 0.14 ^c^
Ash	0.82 ± 0.05 ^d^	4.36 ± 0.43 ^c^	0.32 ± 0.05 ^c^	1.75 ± 0.19 ^b^
Porcine tracheal cartilage sample				

Values are mean ± SD (n = 3). Different lowercase letters (^a–d^) within the same column indicate significant differences among components (p ≤ 0.05).

To determine solid and protein yields, the supernatant was freeze-dried (Christ Gamma 2–16 LSC, Germany). Both yields were expressed relative to the initial pretreated substrate on a dry-solid basis.

(1) $$\text{Solid yield}\;\left(\%\right)=\left(\text{freeze}-\text{dried solid weight}/\text{initial substrate}\;\mathrm{dry}-\text{solid weight}\right)\times 100$$

Protein content of the lyophilized powder was determined by the Kjeldahl method as described above. Protein yield (%) was calculated as:

(2) $$\text{Protein yield}\;\left(\%\right)=\left(\text{protein in hydrolysate}/\text{protein in initial substrate}\right)\times 100$$

The degree of hydrolysis (DH) was determined using the TNBS (trinitrobenzenesulfonic acid) method, which quantifies primary amino groups liberated during proteolysis (Gbogouri et al., 2004). Briefly, 125 μL of sample was mixed with 1 mL of 0.2125 M sodium phosphate buffer (pH 8.2) and 1 mL of 0.1% TNBS solution. The mixture was incubated at 50 °C for 60 min in darkness, after which the reaction was terminated by adding 2 mL of 0.1 M HCl. After cooling to room temperature for 30 min, absorbance was measured at 420 nm. L-leucine (0–2 mM) was used as the standard. The DH (%) was calculated as:

(3) $$\mathrm{DH}\;\left(\%\right)=\left[\left(\alpha -\text{amino groups in sample}-\alpha -\text{amino groups in blank}\right)/\text{total amino groups in substrate}\right]\times 100$$

### 2.4 Bioactivity analysis of hydrolysates

#### 2.4.1 Ferric-reducing antioxidant power (FRAP)

FRAP was determined according to Suwanangul et al. (2025) with slight modifications. The FRAP reagent was freshly prepared by mixing 300 mM acetate buffer (pH 3.6), 10 mM TPTZ (2,4,6-tripyridyl-*s*-triazine) in 40 mM HCl, and 20 mM FeCl₃·6H₂O in a 10:1:1 ratio (*v*/v/v). Sample (10 μL) was mixed with 100 μL FRAP reagent in a 96-well microplate and incubated at room temperature for 15 min. Absorbance was measured at 593 nm using a microplate reader (Varioskan LUX, Thermo Scientific, Vantaa, Finland). Trolox (0–2.5 mM) was used as the standard, and results were expressed as μM Trolox equivalents per mg protein.

#### 2.4.2 Metal chelating activity

Metal chelating activity was assessed following the method of Sangsawad et al. (2016). Sample (100 μL) was mixed with 100 μL of 2 mM FeCl₂ and 200 μL of 5 mM ferrozine in a microplate. After incubation at room temperature for 10 min, absorbance was measured at 562 nm. EDTA (0–1 mM) was used as the standard, and results were expressed as mM EDTA equivalents per mg protein.

#### 2.4.3 2,2′-azino-bis(3-ethylbenzothiazoline-6-sulfonic acid) (ABTS) radical scavenging activity

ABTS radical scavenging was determined according to Boonkong et al. (2026). ABTS radical cation (ABTS•^+^) was generated by reacting 7 mM ABTS with 2.45 mM potassium persulfate (1:1, *v*/v) and allowing the mixture to stand in darkness at room temperature for 12–16 h. The ABTS•^+^ solution was diluted with phosphate buffer (pH 7.4) to an absorbance of 0.70 ± 0.02 at 734 nm. Sample (10 μL) was mixed with 290 μL diluted ABTS•^+^ solution, and absorbance was measured at 734 nm after 6 min. Trolox (0–2.5 mM) was used as the standard, and results were expressed as mM Trolox equivalents per mg protein.

#### 2.4.4 Angiotensin I-converting enzyme (ACE) inhibitory activity

ACE inhibitory activity was measured spectrophotometrically using FAPGG as substrate, following the method of Vermeirssen et al. (2002) with a minor modification. Sample (20 μL) was pipetted into a 96-well microplate, followed by 10 μL of ACE (0.1 U/mL). After pre-incubation at room temperature for 5 min, 70 μL of 0.5 mM FAPGG substrate was added. Absorbance was monitored at 340 nm every 2 min for 30 min at 37 °C using a microplate reader (Varioskan LUX, Thermo Scientific, Vantaa, Finland). The reaction rate (slope) was determined for each sample. ACE inhibitory activity (%) was calculated as:

(4) $$\mathrm{ACE}\;\text{inhibition}\;\left(\%\right)=\left[\left(\text{Slope}\_\text{control}-\text{Slope}\_\text{sample}\right)/\text{Slope}\_\text{control}\right]\times 100$$

#### 2.4.5 Dipeptidyl peptidase-IV (DPP-IV) inhibitory activity

DPP-IV inhibitory activity was assessed using Gly-Pro p-nitroanilide as substrate, following Laosam et al. (2026). Sample (20 μL) was mixed with 10 μL of DPP-IV enzyme (0.05 U/mL) in a 96-well plate and pre-incubated at room temperature for 5 min. Subsequently, 70 μL of 5 mM substrate was added, and absorbance at 405 nm was monitored every 2 min for 30 min at 37 °C using a microplate reader. The reaction rate (slope) was calculated. DPP-IV inhibitory activity (%) was determined as:

(5) $$\mathrm{DPP}-\mathrm{IV}\;\text{inhibition}\;\left(\%\right)=\left[\left(\text{Slope}\_\text{control}-\text{Slope}\_\text{sample}\right)/\text{Slope}\_\text{control}\right]\times 100$$

All hydrolysates were assayed at normalized peptide concentrations (5 mg/mL for antioxidant and DPP-IV inhibitory activities; 0.5 mg/mL for ACE inhibitory activity) to ensure that the observed enzyme-dependent differences reflected specific activity rather than concentration effects.

### 2.5 Ultrafiltration (UF) fractionation

The 4-h Neutrase hydrolysate was subjected to ultrafiltration fractionation. The supernatant (10 mL) was centrifuged through Amicon centrifugal filter units (EMD Millipore, Billerica, MA, USA) with molecular weight cut-off (MWCO) membranes. The sample was first passed through a 10 kDa MWCO membrane at 5000 ×*g* and 4 °C. The retentate (>10 kDa fraction) was washed twice with deionized water and collected. The permeate was subsequently passed through a 3 kDa MWCO membrane under the same conditions to obtain the <3 kDa (permeate) and 3–10 kDa (retentate) fractions. Each fraction was freeze-dried and stored at −20 °C prior to analysis of protein content, total α-amino groups, and bioactive activities. Protein content of all fractions was determined by Bradford assay, and samples were adjusted to normalized protein concentrations (0.5 mg/mL for ACE inhibition; 5 mg/mL for DPP-IV inhibition and antioxidant assays) prior to bioactivity testing. This normalization ensured that observed differences reflect specific activity (bioactivity per unit protein) rather than concentration effects, thereby enabling identification of fractions with low total protein content but high intrinsic bioactivity.

### 2.6 Purification of peptides by Reverse-Phase Flash Chromatography (RP-Flash)

The ultrafiltration fraction with a molecular weight cut-off below 3 kDa (UF3), which exhibited the highest antioxidant and ACE-inhibitory activities, was therefore subjected to Reverse-Phase Flash Chromatography (RP-Flash), adapted from Kong et al. (2020). Purification was performed on a puriFlash® 5.020 system using an Irregular C18 Flash Column. A 3-mL aliquot of sample was injected, and two eluents were employed: solvent A, deionized water containing 0.05% (*v*/v) trifluoroacetic acid (TFA), and solvent B, 100% acetonitrile (ACN). Fractions were collected and the mobile phase was removed by freeze-drying (Freeze Dryer: CHRIST/Gamma 2–16 LSC; Martin Christ Gefriertrocknungsanlagen GmbH, Osterode am Harz, Germany). The dried fractions were subsequently analysed for α-amino group content and evaluated for bioactive activities. Assay concentrations: FRAP, ABTS, metal chelating, and DPP-IV at 5 mg/mL; ACE at 0.5 mg/mL.

### 2.7 Peptide identification by liquid chromatography-tandem mass spectrometry (LC-MS/MS)

Peptide identification was performed through LC-MS/MS analysis of the most bioactive fraction (F5). The F5 fraction from RP-Flash was subjected to de novo peptide sequencing using a capillary LC system (Agilent 1200 series, Agilent Technologies, Santa Clara, CA, USA) coupled to an HCT Ultra PTM Discovery System mass spectrometer (Bruker Daltonics, Bremen, Germany) via an electrospray ionization source operated in positive ion mode. Sample preparation and LC-MS/MS analysis were performed according to Sangsawad et al. (2017). Peptides were separated on a reversed-phase column (Hypersil GOLD, 50 mm × 0.5 mm, 5 μm C18) with a guard column (Hypersil GOLD, 30 mm × 0.5 mm, 5 μm C18) at a flow rate of 300 nL/min. Mass analysis was performed using a Q-TOF detector at 35 kV over a mass range of *m*/*z* 50–1500. Data were processed using the PEAKS scoring system (Bioinformatics Solutions Inc., Waterloo, ON, Canada), which evaluated peptide identification confidence based on MS/MS spectral matching, mass accuracy, and ion coverage. Only high-confidence peptides (PEAKS score > 90%) were selected for subsequent bioinformatics analysis and structure-activity relationship prediction. The identified peptide sequences were chemically synthesized using solid-phase peptide synthesis (SPPS) by GL Biochem (Shanghai) Ltd., Shanghai, China. The synthetic peptides were purified by reversed-phase high-performance liquid chromatography to achieve ≥98% purity and characterized by mass spectrometry (MS) for molecular weight and sequence confirmation before bioactivity evaluation. The ABTS radical scavenging assay was performed at a sample concentration of 5 mg/mL, whereas ACE and DPP-IV inhibitory activities were assayed and expressed as IC_50_ values.

### 2.8 Bioinformatics analysis and structure-activity relationship prediction

Physicochemical and functional properties of peptides were computationally predicted using established bioinformatics tools. Molecular descriptors including molecular weight and isoelectric point were determined via PepDraw (http://pepdraw.com/). Safety assessment employed ToxinPred (https://webs.iiitd.edu.in/raghava/toxinpred/index.html) for toxicity prediction, while PeptideRanker (http://distilldeep.ucd.ie/PeptideRanker/) estimated bioactivity probability based on sequence features. Cell-penetrating potential was evaluated using CPPpred (http://distilldeep.ucd.ie/CPPpred/).

### 2.9 Molecular docking analysis of LGLGADMFHR and QGLPGPGAVAEYT

Molecular docking was adapted from Wen et al. (2025), the crystal structures of human Angiotensin-Converting Enzyme (ACE; PDB 4C2P) and Dipeptidyl Peptidase IV (DPP-IV; PDB 2RIP) were retrieved from the Protein Data Bank. Notably, the mutated ACE residue (R522K) was reverted to the wild-type sequence using Discovery Studio and subsequently energy-minimized prior to the docking procedure to ensure biological accuracy. The minimal energy conformation for the peptide structure was generated using the PEP-FOLD software. Molecular docking studies were subsequently conducted with AutoDock Vina. Prior to docking, the physiological pH and protonation states of the target proteins and peptides were predicted using the PDB2PQR. The active site centre for docking was defined by the coordinates of the existing inhibitor-protein complex. Specifically, a grid box was centred at the coordinates x = −13.15, y = 8.15, and z = −23.05 for ACE and x = 62.44, y = 52.77, and z = 84.52 for DPP-IV. The resulting docking complexes and interactions were then visualized and analysed with UCSF Chimera and Discovery Studio.

### 2.10 Simulated gastrointestinal digestion

The stability and transformation of the 4-h Neutrase hydrolysate during gastrointestinal digestion were evaluated using a simplified in vitro digestion model (standardised INFOGEST) adapted from Minekus et al. (2014). The digestion protocol consisted of two sequential phases. Gastric phase, hydrolysate (10 mL) was adjusted to pH 2.0 with 6 M HCl, and pepsin (2000 U/mL protein) was added. The mixture was incubated at 37 °C under continuous shaking (100 rpm) for 2 h. Intestinal phase, after the gastric phase, the pH was adjusted to 7.0 with 1 M NaHCO₃. Pancreatin (100 U trypsin activity/mL protein) was added, and the mixture was incubated at 37 °C with shaking (100 rpm) for an additional 2 h (cumulative time: 4 h total digestion).

Aliquots were collected at 0 h (before digestion), 2 h (end of gastric phase), and 4 h (end of intestinal phase). Enzymes were immediately inactivated by heating to 90 °C for 10 min. Samples were centrifuged at 5000 ×*g* for 10 min at 4 °C, and supernatants were stored at −20 °C for subsequent analysis of peptide content, molecular weight distribution, and bioactive activities.

### 2.11 Statistical analysis

All experiments were performed in triplicate, and results are expressed as mean ± standard deviation (SD). Data were analysed using one-way analysis of variance (ANOVA) followed by Duncan's multiple range test to identify significant differences among means. Statistical significance was set at *p* ≤ 0.05. Analyses were conducted using SPSS Statistics software version 26.0 (IBM Corporation, Armonk, NY, USA). In addition, Pearson correlation coefficients (r) were computed using SciPy v1.11 across UF fractions to assess directional relationships between molecular weight and bioactivity indicators.

## 3 Results and discussion

### 3.1 Proximate composition of alkaline-pretreated porcine tracheal cartilage

The proximate composition of porcine tracheal cartilage before and after alkaline pretreatment is presented in Table 1. In the untreated tissue, protein and total lipid represented 76.24% and 19.40% of the dry matter (14.31% and 3.67% on a wet-weight basis), respectively. Alkaline pretreatment significantly enriched the protein fraction to 97.11% (dry basis; 17.97% wet basis) while reducing the total lipid content to 1.15% (dry basis; 0.21% wet basis) and lowering the ash content from 4.36% to 1.75% (dry basis), with the protein, lipid, and ash fractions differing significantly between the two samples (*p* ≤ 0.05). The high protein content confirms effective enrichment of the collagen-rich protein fraction through alkaline pretreatment, which removed the majority of the lipids and non-collagenous components, corresponding to a loss of approximately 21% of the initial dry solids and establishing this material as an appropriate substrate for enzymatic hydrolysis targeting bioactive peptide generation. This compositional profile aligns with expectations for cartilaginous tissue, in which structural proteins dominate the organic matrix (Cui et al., 2024; Kim et al., 2025).

### 3.2 Enzymatic hydrolysis and activity-dependent bioactivity relationships

**Fig. 1:**
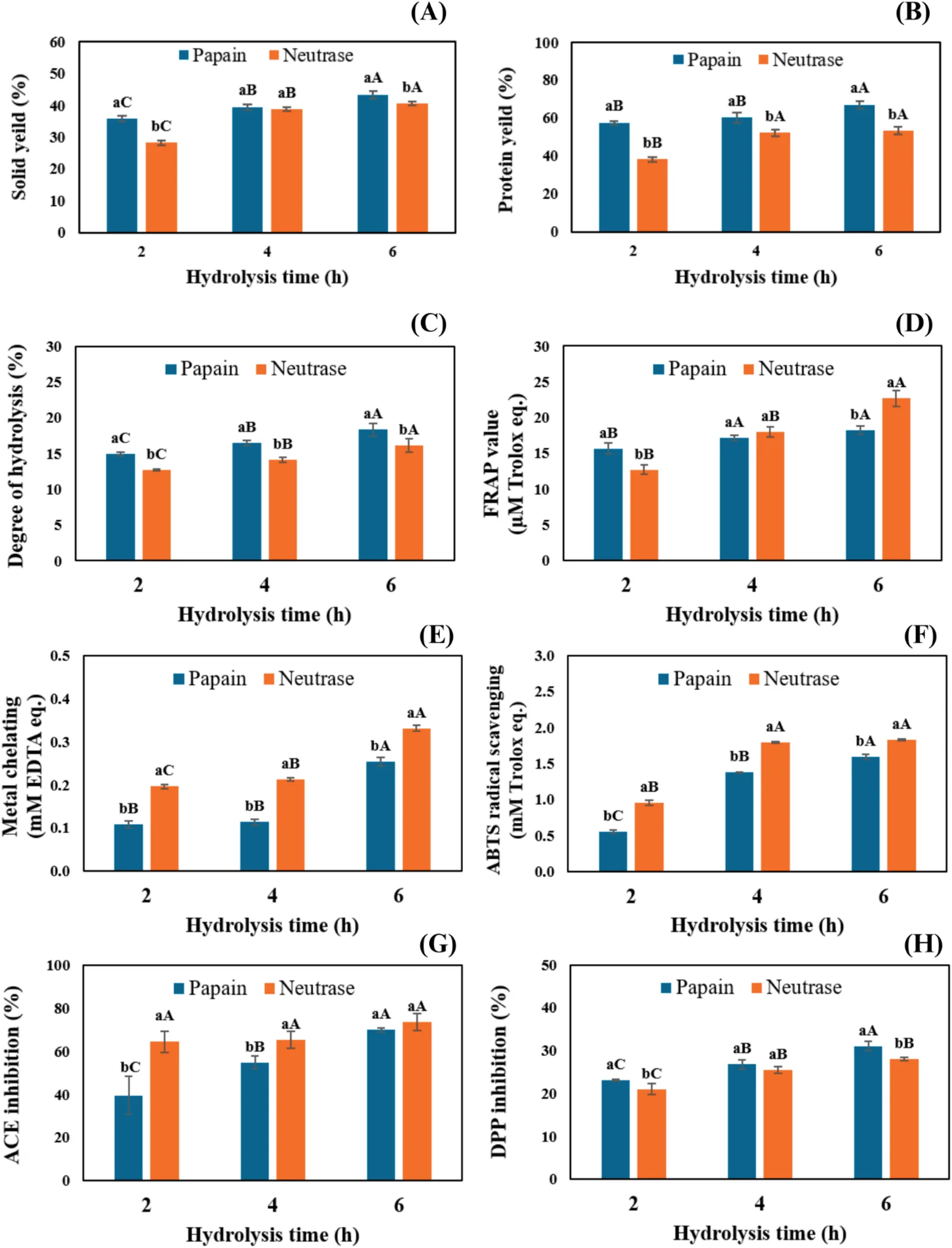
Comparative evaluation of Papain and Neutrase hydrolysis of porcine tracheal cartilage at 2, 4, and 6 h. **(A)** Solid yield (%). **(B)** Protein yield (%). **(C)** Degree of hydrolysis (DH, %). **(D)** Ferric-reducing antioxidant power (FRAP, μM Trolox equivalents). **(E)** Metal chelating activity (mM EDTA equivalents). **(F)** ABTS radical scavenging activity (mM Trolox equivalents). **(G)** Angiotensin I-converting enzyme (ACE) inhibitory activity (%). **(H)** Dipeptidyl peptidase-IV (DPP-IV) inhibitory activity (%). Different lowercase letters (^a–b^) indicate significant differences between enzymes at the same time point (*p* ≤ 0.05). Different uppercase letters (^A–C^) indicate significant differences among time points within the same enzyme (p ≤ 0.05). Assay concentrations: FRAP, ABTS, metal chelating, and DPP-IV at 5 mg/mL; ACE at 0.5 mg/mL. Values represent mean ± SD (*n* = 3).

Enzymatic hydrolysis of alkaline-pretreated porcine tracheal cartilage proceeded successfully with both Papain and Neutrase, yielding time-dependent changes in solid yield, protein yield, DH, and bioactivity **(**Fig. 1**)**. Solid yield increased progressively with hydrolysis time for both enzymes, with Papain achieving slightly higher yields (43.62% at 6 h) compared to Neutrase (39.66% at 6 h; Fig. 1A**)**. Protein yield (recovery) exhibited a similar trend, with Papain and Neutrase achieving 60.44% and 52.34% soluble protein recovery, respectively, at 6 h (p ≤ 0.05). These protein yields are consistent with values reported for commercial protease hydrolysis of collagen-rich porcine by-products using Neutrase, Papain, and related proteases under comparable conditions (Damrongsakkul et al., 2008; Hong et al., 2019) (Fig. 1B). Papain hydrolysis yielded significantly higher DH values than Neutrase at all time points, reaching 18.65% at 6 h compared to 16.50% for Neutrase (*p* ≤ 0.05; Fig. 1C**)**.

The consistently higher DH values obtained with Papain hydrolysis reflect fundamental differences in enzyme substrate specificity. Papain, a cysteine endopeptidase, exhibits broad substrate specificity with preference for hydrophobic residues (Phe, Leu, Val), enabling extensive cleavage within collagen's Gly-X-Y sequences. In contrast, Neutrase, a Bacillus-derived neutral metalloprotease, shows more restrictive specificity, preferentially cleaving Leu-X and Phe-X bonds with limited activity toward proline-rich collagen sequences (Damrongsakkul et al., 2008; Hong et al., 2019). Consequently, while both enzymes operated efficiently under the experimental conditions employed, Papain's relaxed substrate specificity facilitated hydrolysis of a greater number of peptide bonds, resulting in elevated DH and correspondingly higher protein solubilization. Both enzymes demonstrated plateau behaviour beyond 4 h, with DH increasing only marginally between 4 h and 6 h **(**Fig. 1C**)**. This plateau reflects declining enzyme activity as substrate becomes depleted and product inhibition sets in (Gbogouri et al., 2004; Moreno-Mariscal et al., 2025). The 4-h timepoint therefore represents an optimal balance between hydrolysis extent and processing efficiency.

Bioactivity screening revealed patterns warranting careful mechanistic interpretation. Antioxidant activities demonstrated distinct enzyme-dependent patterns across multiple assays. For FRAP **(**Fig. 1D**)**, Neutrase hydrolysates exhibited superior ferric-reducing capacity at 6 h (22.68 μM Trolox equivalents) compared to Papain (18.23 μM Trolox equivalents; *p* ≤ 0.05), despite Papain achieving higher DH. Metal chelating activity showed an even more pronounced difference **(**Fig. 1E**)**, with Neutrase at 6 h reaching 0.33 mM EDTA equivalents versus Papain's 0.25 mM EDTA equivalents (p ≤ 0.05). Similarly, ABTS radical scavenging activity **(**Fig. 1F**)** revealed Neutrase's superiority, achieving 1.85 mM Trolox equivalents at 6 h compared to Papain's 1.56 mM Trolox equivalents (p ≤ 0.05). These convergent patterns across multiple antioxidant assays suggest that Neutrase's selective proteolysis generates peptide populations enriched in structural features favourable for antioxidant functionality, particularly specific sequence motifs containing hydrophobic and electron-donating residues that contribute to mechanisms such as electron donation, hydrogen atom transfer, and metal chelation (Paksin et al., 2026; Purba & Paengkoum, 2022).

For ACE inhibitory activity **(**Fig. 1G**)**, Neutrase hydrolysates demonstrated superior performance despite lower DH values. At 6 h, Neutrase hydrolysate exhibited 77.42% ACE inhibition compared to Papain's 70.46% (*p* ≤ 0.05). This inverse relationship indicates that ACE inhibitory potency depends on generation of specific peptide sequences with appropriate amino acid composition and conformational properties rather than on total extent of proteolytic degradation. Neutrase's more restricted substrate specificity apparently gives rise to cleavage patterns that yield functionally optimal sequences for ACE inhibition. The mechanistic basis for this selectivity-driven bioactivity enhancement is rooted in firm structural principles. The ACE active site comprises a relatively confined substrate-binding pocket with specific recognition requirements for optimal inhibitor binding. Structural studies demonstrate that the substrate-binding pocket harbors appreciable hydrophobic character (Taghizadeh et al., 2025). Peptides lacking appropriate residues at key positions exhibit reduced inhibitory potency regardless of concentration. Small molecular size may facilitate effective ACE inhibition via penetration into the confined geometry of the substrate-binding cleft. Neutrase's selective proteolysis appears to generate peptide populations enriched in these structurally favourable characteristics. Papain's broader specificity, while achieving higher overall hydrolysis, may cleave bioactive sequence motifs or generate peptides exceeding optimal size ranges for ACE active site accessibility. The convergence between antioxidant activity patterns (Fig. 1D-F) and ACE inhibition (Fig. 1G) suggests shared structural requirements, where specific sequence motifs containing hydrophobic residues and appropriate molecular size contribute to both functionalities.

For DPP-IV inhibition **(**Fig. 1H**)**, a different pattern emerged. Papain hydrolysate with higher hydrolysis extent exhibited superior DPP-IV inhibition compared to Neutrase hydrolysate. At 6 h, Papain reached 31.12 ± 1.15% inhibition versus Neutrase's 27.89 ± 0.89% (*p* ≤ 0.05). This pattern supports the interpretation that DPP-IV inhibition requires broader peptide diversity and potentially smaller average molecular weight arising from more extensive hydrolysis. More importantly, the divergent patterns across these bioactivities demonstrate that different enzyme targets operate under distinct structure-activity requirements rather than conforming to a single universal principle relating hydrolysis extent to bioactivity outcomes (Moreno-Mariscal et al., 2025). The mechanistic differences are substantial. DPP-IV, a serine protease with catalytic mechanism and active site architecture differing markedly from the zinc metallopeptidase ACE, imposes different structural constraints on inhibitor binding (Pantaleão et al., 2018). The enzyme may possess less stringent sequence requirements, or may rely on the presence of multiple moderately active peptides that contribute to inhibition through additive or synergistic effects. The more extensive peptide generation through Papain's broad-specificity proteolysis therefore proves advantageous for DPP-IV inhibition, enabling statistical probability that the hydrolysate harbors multiple sequences capable of binding to the active site.

Overall, these findings demonstrate that enzyme selection should be guided by target-specific structure–activity requirements rather than by degree of hydrolysis or protein yield alone, since high hydrolysis extent may favour some bioactivities while compromising others, consistent with patterns reported across protein hydrolysate systems (Laosam et al., 2024; Moreno-Mariscal et al., 2025).

### 3.3 Molecular weight distribution and activity-dependent size relationships

**Fig. 2:**
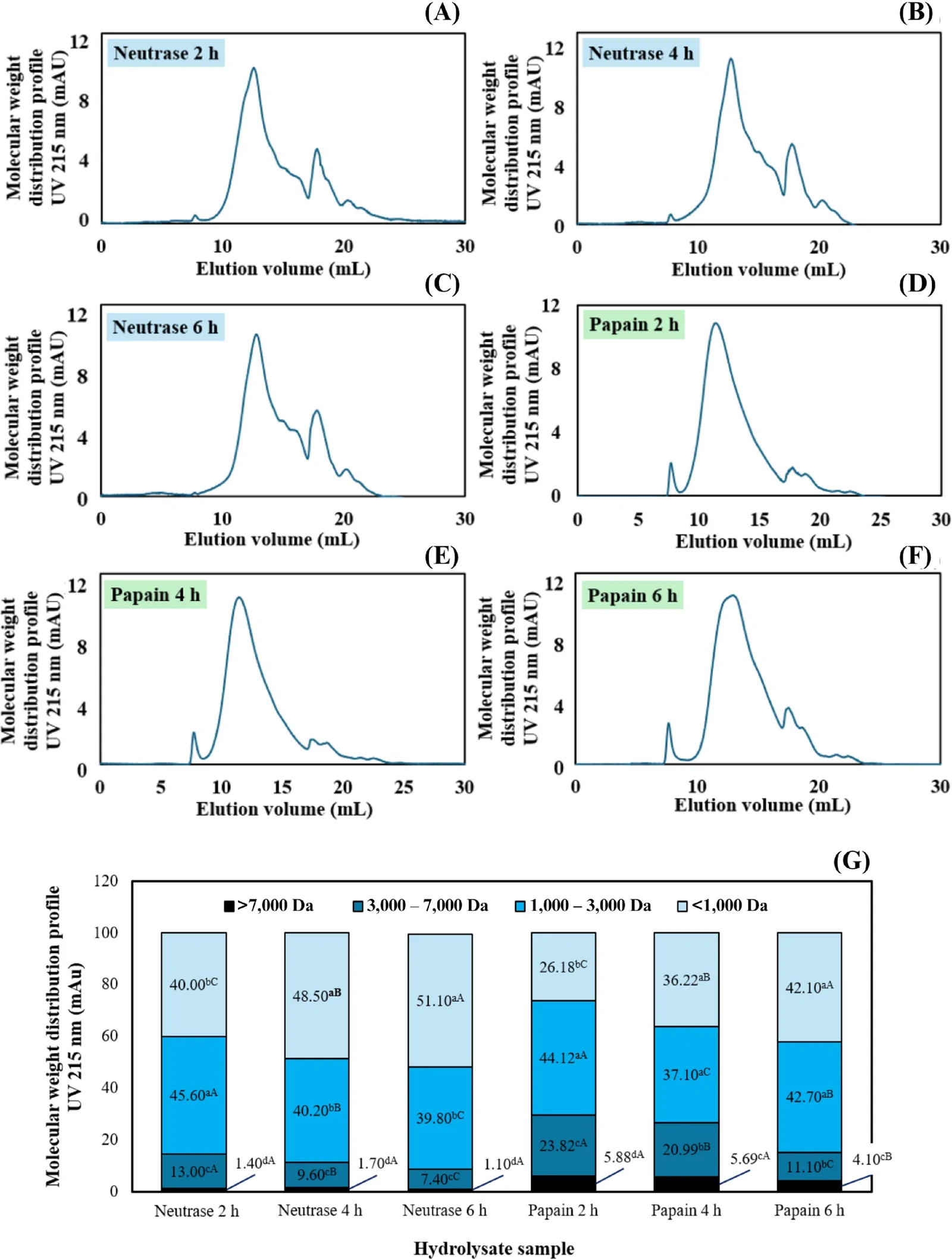
Molecular weight distribution of peptides generated by Papain and Neutrase hydrolysis. **(A–F)** Size exclusion chromatography profiles showing peptide distribution after 2, 4, and 6 h hydrolysis. **(G)** Percentage distribution of peptide molecular weight ranges (<1000,1000−3000,3000−7000,>7000 Da). Different lowercase letters (^a-d^) indicate significant differences among molecular weight fractions within the same enzyme and time point (p ≤ 0.05). Different uppercase letters (^A-C^) indicate significant differences for the same molecular weight fraction across time points within the same enzyme (p ≤ 0.05). Values represent mean ± SD (n = 3).

Size exclusion chromatography revealed distinct molecular weight profiles between Papain and Neutrase hydrolysates across hydrolysis times **(**Fig. 2A-F**).** Despite achieving lower overall DH compared to Papain, Neutrase hydrolysate demonstrated enrichment of peptides below 3 kDa molecular weight. Neutrase hydrolysate contained a 1.2-fold higher proportion of peptides below 3 kDa than Papain hydrolysate (Fig. 2G; p ≤ 0.05). This indicates that Neutrase's substrate specificity steers proteolysis toward generation of predominantly small peptides through selective cleavage at positions that liberate low molecular weight fragments. Papain's broader specificity, while achieving higher total percentage of cleaved bonds, generates a more heterogeneous size distribution spanning wider molecular weight ranges. This size distribution characteristic underlies the superior ACE inhibitory activity of the Neutrase hydrolysate discussed in Section 3.2. Systematic analysis of bioactive peptides has established that peptides below 3 kDa demonstrate enhanced bioactivity for both antihypertensive and antioxidant functions, with smaller molecular weight facilitating cellular absorption and target site accessibility (Tang et al., 2025). Small peptides confer structural advantages for penetrating the confined geometry of the ACE binding pocket. Larger peptides may encounter steric exclusion even when harboring bioactive sequence motifs.

The convergence of evidence from comparative enzyme evaluation, size distribution analysis and subsequent ultrafiltration fractionation results points toward a coherent mechanistic interpretation. Neutrase's substrate specificity directs proteolysis toward size distributions enriched in small peptides with favourable size-dependent binding characteristics for ACE inhibition. This mechanism accounts for the apparent paradox of lower overall hydrolysis extent yielding superior bioactivity for this particular enzyme target (Khongla et al., 2026). The data underscore that simple degree of hydrolysis measurements, while useful for tracking proteolysis progress, cannot substitute for comprehensive characterization of the resulting peptide populations' functional properties.

Taken together, the enzyme-screening and molecular-weight results identified the 4-h Neutrase hydrolysate as the most suitable material for downstream fractionation. Neutrase consistently outperformed Papain in ACE inhibition and antioxidant activity (Section 3.2) and generated a higher proportion of peptides below 3 kDa (Section 3.3), the size range associated with the target bioactivities. Although several parameters continued to rise marginally up to 6 h, the solid yield, protein yield, ACE inhibition (73.5% at 4 h versus 77.42% at 6 h), ABTS radical scavenging, and proportion of <3 kDa peptides had, by 4 h, already reached values close to those at 6 h (Fig. 1, Fig. 2), indicating a plateau in hydrolysis beyond 4 h. The 4-h time point was therefore selected because it retained near-maximal yield and bioactivity while shortening the hydrolysis by 2 h, improving processing efficiency without a meaningful loss of performance. Accordingly, the 4-h Neutrase hydrolysate was carried forward for ultrafiltration fractionation.

### 3.4 Ultrafiltration fractionation and size-dependent bioactivity

**Fig. 3:**
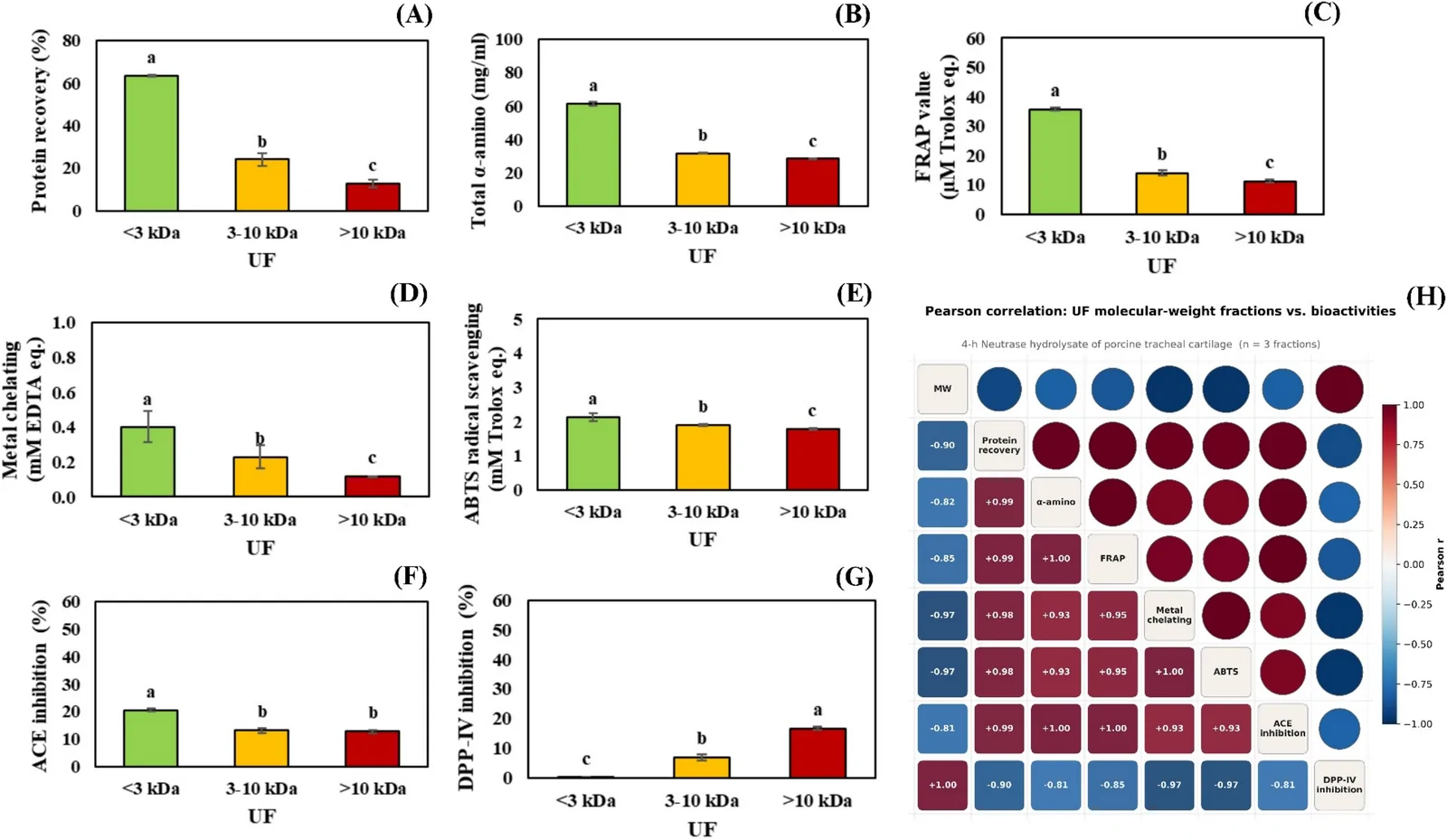
Peptide content, protein recovery, and bioactivities of ultrafiltration (UF) fractions from 4-h Neutrase hydrolysate of porcine tracheal cartilage. **(A)** Protein recovery (%). **(B)** Total α-amino groups (mg/mL). **(C)** Ferric-reducing antioxidant power (FRAP, μM Trolox equivalents). **(D)** Metal chelating activity (mM EDTA equivalents). **(E)** ABTS radical scavenging activity (mM Trolox equivalents). **(F)** ACE inhibitory activity (%). **(G)** DPP-IV inhibitory activity (%). **(H)** Pearson correlation matrix of molecular weight and the measured parameters. Different letters (^a-c^) indicate significant differences among fractions (*p* ≤ 0.05). Assay concentrations: FRAP, ABTS, metal chelating, and DPP-IV at 5 mg/mL; ACE at 0.5 mg/mL. Values represent mean ± SD (*n* = 3).

Ultrafiltration fractionation of the 4-h Neutrase hydrolysate into three molecular weight ranges revealed clear size-dependent bioactivity patterns **(**Fig. 3**)**. All ultrafiltration fractions were assayed at normalized protein concentrations (0.5 mg/mL for ACE inhibition; 5 mg/mL for DPP-IV inhibition and antioxidant assays) to ensure that observed differences in bioactivity reflect variations in specific activity rather than concentration effects. Protein concentrations were determined by Bradford assay and adjusted to equivalent values prior to bioactivity testing.

Protein recovery analysis **(**Fig. 3A**)** showed that the <3 kDa fraction (UF3) contained the highest protein content (63.55%), followed by the 3–10 kDa fraction (UF2, 24.13%) and > 10 kDa fraction (UF1, 12.32%), confirming successful enrichment of small peptides through Neutrase hydrolysis. Total α-amino group content followed a similar distribution pattern **(**Fig. 3B**)**, with UF3 exhibiting the highest value (62.18 mg/mL), indicating abundant free amino termini from extensive peptide bond cleavage in this low molecular weight fraction.

Size-dependent antioxidant activities demonstrated consistent superiority of smaller peptides. UF3 achieved the highest FRAP value (35.79 μM Trolox equivalents; Fig. 3C), representing 2.5-fold and 3.2-fold increases compared to UF2 (14.26 μM Trolox equivalents) and UF1 (11.13 μM Trolox equivalents), respectively (*p* ≤ 0.05). Metal chelating activity exhibited the same trend **(**Fig. 3D**)**, with UF3 showing 0.40 mM EDTA equivalents versus 0.23 mM (UF2) and 0.11 mM (UF1; p ≤ 0.05). ABTS radical scavenging activity also favoured smaller peptides **(**Fig. 3E**)**, with UF3 achieving 2.14 mM Trolox equivalents compared to 1.93 mM (UF2) and 1.78 mM (UF1; p ≤ 0.05). The pronounced enrichment of bioactivity in the <3 kDa fraction parallels findings for ultrafiltration-fractionated hydrolysates from other collagen-rich substrates, in which low-molecular-weight fractions consistently concentrate ACE-inhibitory and antioxidant activities (Tang et al., 2025; W. Hu et al., 2025).

ACE inhibitory activity showed the strongest size-dependence **(**Fig. 3F**)**. At the standardised concentration (0.5 mg/mL), UF3 achieved 20.59% inhibition versus 13.27% (UF2) and 12.82% (UF1; p ≤ 0.05), demonstrating 1.6-fold higher specific activity than larger fractions. This enhanced specific activity likely stems from improved accessibility to the ACE active site (W. Hu et al., 2025). This outcome confirms the size-activity relationship established in Section 3.2, where Neutrase's preferential generation of small peptides correlated with superior ACE inhibition. The 1.6-fold higher ACE specific activity of UF3 relative to larger fractions indicates that the size–activity relationship of tracheal cartilage peptides conforms to the general behaviour of collagen-derived systems (Khongla et al., 2026), despite its distinct Type II collagen origin.

In contrast to other bioactivities, DPP-IV inhibition exhibited an inverse size-dependence **(**Fig. 3G**)**, with UF1 showing the highest inhibition (16.82%), followed by UF2 (6.94%) and UF3 (1.30%; p ≤ 0.05). This pattern suggests that DPP-IV accommodates or even favors larger peptide substrates, possibly due to extended substrate-binding sites beyond the catalytic pocket that benefit from additional peptide-enzyme contacts.

The convergence of superior ACE inhibition and multifaceted antioxidant activities in the UF3 fraction aligns with cardiovascular health applications (Khongla et al., 2026), where blood pressure regulation and oxidative stress mitigation act synergistically. Based on these results, fraction UF3 was selected for further purification through reversed-phase flash chromatography and subsequent peptide identification.

To quantitatively support these size-dependent patterns, Pearson correlation coefficients were computed across the three UF fractions **(**Fig. 3H**)**. Molecular weight showed a strong positive correlation with DPP-IV inhibition (r = +1.00) and strong negative correlations with ABTS scavenging (*r* = −0.97), metal chelating (r = −0.97), protein recovery (*r* = −0.90), FRAP (*r* = −0.85), total α-amino content (*r* = −0.82), and ACE inhibition (*r* = −0.81). Conversely, protein recovery and total α-amino content were strongly positively correlated with FRAP, metal chelating, ABTS, and ACE inhibition (*r* = 0.98–0.99). Given the limited number of fractions (*n* = 3), these correlation coefficients are interpreted as directional trends rather than tests of statistical significance, but they consistently confirm that smaller peptides (<3 kDa) drive multifunctional antioxidant and ACE-inhibitory activities, whereas DPP-IV inhibition is uniquely associated with larger peptide species.

### 3.5 Reverse-phase flash chromatography (RP-Flash) and hydrophobicity-bioactivity relationships

**Fig. 4:**
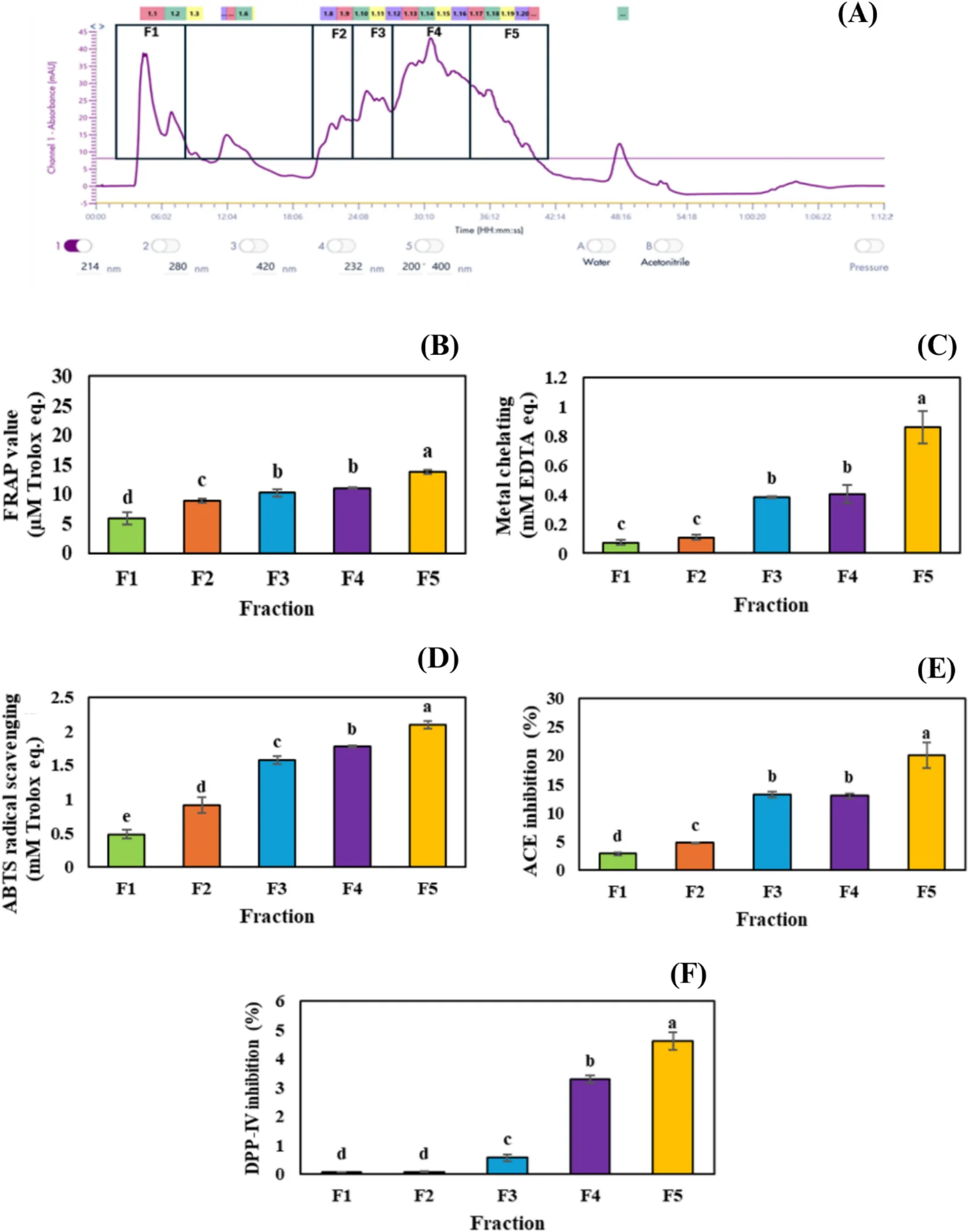
Bioactivities of reverse phase flash chromatography (RP-Flash) for fractions F1–F5. **(A)** Representative chromatogram indicating collection windows for fractions F1–F5. **(B)** Ferric-reducing antioxidant power (FRAP). **(C)** Metal chelating activity. **(D)** ABTS radical scavenging activity. **(E)** ACE inhibitory activity. **(F)** DPP-IV inhibitory activity. Different letters (^a-e^) indicate significant differences among fractions (*p* ≤ 0.05). Assay concentrations: FRAP, ABTS, metal chelating, and DPP-IV at 5 mg/mL; ACE at 0.5 mg/mL. Values represent mean ± SD (*n* = 3).

The <3 kDa ultrafiltration fraction of our study, having demonstrated superior bioactivities, was subjected to reversed-phase flash chromatography to further fractionate peptides based on hydrophobic character (Fig. 4**)**. Stepwise gradient elution with increasing acetonitrile concentration (0–30%) yielded five distinct fractions designated F1-F5 (Fig. 4A**)**. The chromatographic separation achieved clear resolution of peptide populations with distinct hydrophobicity profiles, as evidenced by differential elution times correlating with acetonitrile concentration requirements for desorption from the nonpolar stationary phase. Fractions F4 and F5 contain more hydrophobic peptides than earlier fractions, as their stronger interaction with the C18 stationary phase requires higher acetonitrile concentrations for elution. Consistent with the scientific consensus, this separation principle enables systematic investigation of hydrophobicity-bioactivity relationships independent of molecular weight effects, since all fractions originate from the size-controlled <3 kDa pool (W. Hu et al., 2025; Moreno-Mariscal et al., 2025; Taghizadeh et al., 2025).

Bioactivity assessment of the five fractions revealed progressive enhancement of antioxidant activities from F1 to F5. Antioxidant capacity showed the greatest enhancement in F5, consistent with the positive correlation between moderate hydrophobicity and antioxidant activity (Li et al., 2022; Tang et al., 2025). FRAP values for F5 reached 13.77 μM Trolox equivalents (Fig. 4B), representing a 35% increase over F3 (10.18 μM). Metal chelating activity (Fig. 4C) and ABTS radical scavenging capacity (Fig. 4D) followed similar trends, with F5 achieving 0.86 mM EDTA equivalents and 2.09 mM Trolox equivalents respectively. The antioxidant enhancement with increasing hydrophobicity suggests that hydrophobic peptides may possess multifunctional bioactivities through complementary mechanisms. The antioxidant mechanism likely involves aromatic amino acids, particularly phenylalanine, tyrosine, and tryptophan, whose hydrophobic character contributes both to ACE inhibition (through hydrophobic binding interactions) and to electron-donating or radical-scavenging capacity. Aromatic residues can donate electrons to reduce ferric ions (FRAP mechanism) or directly scavenge free radicals through formation of stabilized aromatic radical intermediates (ABTS mechanism). The moderate correlation between hydrophobicity and metal chelation (Fig. 4C) may reflect that effective metal coordination depends primarily on spatial arrangement of donor atoms rather than overall hydrophobic character (Purba et al., 2026; Udenigwe & Aluko, 2012), though hydrophobic residues adjacent to chelating groups could influence conformational flexibility affecting coordination geometry.

ACE inhibitory activity also correlated strongly with increasing hydrophobicity (Fig. 4E**)**. Fraction F5, eluting at 25–30% acetonitrile representing the most hydrophobic peptide population, demonstrated the highest ACE inhibition (20.03% at 0.5 mg/mL), significantly surpassing fractions F1 (2.82%), F2 (4.71%), F3 (13.18%), and F4 (12.95%), with F5 differing significantly from all other fractions (*p* ≤ 0.05). This systematic gradient spanning 17.2 percentage points from least to most hydrophobic fractions establishes hydrophobicity as a critical determinant of ACE inhibitory potency. The observation that fraction F5 exhibited the highest ACE inhibitory activity among all chromatographic fractions reveals an important structure-activity relationship, indicating clearly that ACE inhibition is favoured by peptides combining small molecular weight (<3 kDa, established through ultrafiltration) with high hydrophobicity (demonstrated through reversed-phase separation). These results confirm the size and hydrophobicity requirements for ACE inhibitory activity established in 3.2, 3.3. This dual structural requirement holds on mechanistic grounds rooted in the three-dimensional architecture of the ACE active site. Small molecular size enables penetration into the confined geometry of the substrate-binding pocket, and hydrophobic residues facilitate binding through nonpolar interactions with hydrophobic amino acids lining the substrate-binding subsites within the active site cleft (W. Hu et al., 2025). Crystallographic and molecular dynamics studies of ACE demonstrate that the substrate-binding pocket possesses substantial hydrophobic character, particularly within the S1 and S1’ subsites (Natesh et al., 2003; Wen et al., 2025). These subsites contain hydrophobic amino acids including valine, phenylalanine, and leucine that create favourable binding environments for peptide inhibitors exhibiting complementary hydrophobic characteristics. Peptides lacking adequate hydrophobic content, even if possessing appropriate size and sequence motifs, may exhibit diminished binding affinity due to insufficient van der Waals interactions with these hydrophobic subsites, whereas highly hydrophobic peptides of excessive molecular weight may encounter steric exclusion from the confined binding pocket despite possessing favourable hydrophobic character. The convergence of small size and substantial hydrophobicity in F5 peptides therefore represents an optimal combination maximizing both accessibility to the active site (size requirement) and binding affinity once positioned within the pocket (hydrophobicity requirement). This principle aligns with established structure-activity relationships for ACE inhibitory peptides from diverse protein sources (Antony et al., 2024; Taghizadeh et al., 2025).

DPP-IV inhibition demonstrated moderate hydrophobicity dependence (Fig. 4F), with F5 achieving 4.60 ± 0.31% inhibition versus 0.57 ± 0.12% for F3 and 0.06 ± 0.01% for F1 (p ≤ 0.05). The less dramatic hydrophobicity effect for DPP-IV compared to ACE suggests different active site characteristics and inhibitor binding requirements between these two enzymes. DPP-IV recognizes inhibitors primarily through N-terminal sequence specificity, particularly requiring proline at position 2, with hydrophobic interactions playing a secondary role (Taghizadeh et al., 2025; Wen et al., 2025). This mechanistic difference explains why DPP-IV inhibition shows less pronounced hydrophobicity dependence compared to ACE where hydrophobic interactions constitute a primary binding determinant. Based on superior ACE inhibitory activity combined with substantial multifunctional antioxidant capacity, fraction F5 was selected for comprehensive peptide sequence identification through liquid chromatography tandem mass spectrometry analysis. This fraction combines the small molecular weight (<3 kDa) established through ultrafiltration and the high hydrophobicity demonstrated through reversed-phase chromatography, representing the structural convergence that the preceding fractionation experiments identified as optimal for ACE inhibitory potency. These characteristics are predicted to maximize ACE inhibitory potency based on structure-activity principles derived from both our experimental fractionation results and established literature on collagen-derived bioactive peptides across diverse sources.

### 3.6 Peptide sequence identification and in silico characterization

LC-MS/MS analysis of fraction F5 identified eight peptide sequences ranging from 870 to 1565 Da (Table 2). All sequences originated from Type II collagen (UniProt database), consistent with the predominant protein composition of tracheal cartilage established through proximate analysis. The identified sequences exhibited structural features characteristic of bioactive peptides derived from collagen sources. Multiple proline residues appeared throughout most sequences, reflecting collagen's high proline content approaching 20% of total amino acid composition (Vermeirssen et al., 2002). Proline residues confer resistance to proteolytic degradation through their cyclic pyrrolidine ring structure, which restricts peptide bond accessibility to certain classes of proteases. Hydrophobic residues including leucine, valine, phenylalanine, and alanine distributed across the sequences. Several peptides contained aromatic amino acids (phenylalanine, tyrosine) potentially contributing to antioxidant capacity through hydrogen atom donation mechanisms and to ACE inhibition through hydrophobic interactions within the enzyme active site (Li et al., 2022).

**Table 2:** Amino acid sequences of peptides from fraction F5 and their characteristics.⁎, B, PC

No.	Identified peptide	Mass (Da)	pI	Peptide ranker	CPPpred	Toxicity	ABTS radical scavenging (mM Trolox equivalents)	ACE inhibition (IC_50_, mM)	DPP-IV inhibition (IC_50_, mM)	Peptide fragment after in silico gastrointestinal digestion^⁎PC^	Reported bioactivity of the peptide fragments^⁎ B^
1	ESLGLGQAP	870.44	3.01	0.18	0.13	NT	2.26^e^	NI	NI	**ES**-L-G-L-GQAP	(ES) DPP-IV-inhibitory
2	GPHAPHNGPR	1038.51	11.13	0.82	0.16	NT	3.86^c^	0.80^e^	NI	GPH-APH-N-**GPR**	(GPR) AC*E*-inhibitory, DPP-IV-inhibitory
3	SSMLGLMGGPV	1047.51	5.54	0.52	0.11	NT	3.27^d^	0.98^d^	6.09^a^	SSM-L-G-L-M-GGPV	No report
4	LGFGADRAGPQ	1087.54	6.74	0.38	0.17	NT	5.64^a^	5.39^b^	NI	L-G-F-GADR-AGPQ	No report
5	LGLGADMFHR	1115.55	7.89	0.66	0.16	NT	2.46^e^	0.52^f^	NI	L-G-L-GADM-F-H-R	No report
6	KREGYTLEYT	1258.62	6.66	0.09	0.21	NT	1.27^f^	NI	4.24^b^	K-R-EGY-T-L-**EY**-T	(EY) ACE-inhibitory, DPP-IV-inhibitory
7	QGLPGPGAVAEYT	1258.62	3.08	0.37	0.14	NT	2.27^e^	7.02^a^	1.84^d^	QGL-PGPGAVAEY-T	No report
8	KPWEADWKELYT	1564.75	4.33	0.64	0.24	NT	4.21^b^	1.23^c^	2.64^c^	K-**PW**-EADW-K-E-L-Y-T	(PW) ACE-inhibitory, DPP-IV-inhibitory, Antioxidant

Significantly different ^a-f^ mean values are denoted by distinct superscripts within the same column.

The ABTS radical scavenging assay was performed at a sample concentration of 5 mg/mL.

IC_50_ values for ACE and DPP-IV inhibition are expressed in mM. “NI” denotes no inhibition reaching 50% at the highest concentration tested (10 mg/mL).

⁎ Data obtained from the Bioactive Peptide Database: http://www4g.biotec.or.th/FeptideDB/enzyme_digestion.php

B Data obtained from BIOPEP database: http://www.uwm.edu.pl/biochemia/index.php/pl/biopep

PC means peptide was simulated to be digested by pepsin followed by chymotrypsin.

Computational structure-activity prediction tools provided preliminary assessment of bioactive potential for the identified sequences (Table 2). PeptideRanker analysis, which quantifies bioactivity probability based on established sequence-function databases, yielded scores ranging from 0.09 to 0.82. Six of eight sequences achieved scores above 0.30, indicating substantial bioactive potential relative to random peptide populations. GPHAPHNGPR received the highest PeptideRanker score (0.82), suggesting strong likelihood of biological activity. In contrast, KREGYTLEYT scored lowest (0.09), indicating lower predicted bioactivity despite containing potentially functional residues. This distribution proves typical for peptide mixtures derived from enzymatic hydrolysis, where only specific sequences harbor structural features conferring potent bioactivity while others contribute minimally to overall functional properties.

Following identification, all eight peptides were chemically synthesized and experimentally evaluated to validate their bioactive properties. For ACE inhibitory activity, IC_50_ values for the active peptides ranged from 0.52 mM to 7.02 mM, while the other two sequences showed no inhibition reaching 50% at the highest concentration tested (10 mg/mL) **(**Table 2**)**. The synthesized peptide LGLGADMFHR demonstrated the most potent ACE inhibition (IC_50_ = 0.52 mM), attributed to C-terminal hydrophobic residues (methionine, phenylalanine, histidine, arginine) and metal-coordinating aspartic acid that are known to be associated with effective ACE inhibition. GPHAPHNGPR also showed strong experimental potency (IC_50_ = 0.80 mM), likely stemming from its high PeptideRanker score and proline-rich composition. Two peptides (ESLGLGQAP and KREGYTLEYT) showed no inhibition reaching 50% at the highest concentration tested (10 mg/mL), indicating negligible ACE inhibitory activity.

For DPP-IV inhibition, IC_50_ values for the active peptides ranged from 1.84 mM to 6.09 mM, while four sequences showed no inhibition reaching 50% at the highest concentration tested (10 mg/mL) **(**Table 2**)**. The synthesized peptide QGLPGPGAVAEYT showed the most potent DPP-IV inhibition (IC_50_ = 1.84 mM), likely reflecting its multiple internal proline residues (positions 4 and 6), which impose conformational rigidity favourable for accommodation within the DPP-IV active site. Although DPP-IV characteristically recognizes substrates bearing proline at the penultimate (P1) position, inhibitory peptides frequently engage the active site through proline-rich motifs that provide complementary structural constraints rather than strict N-terminal X-Pro recognition. KPWEADWKELYT and KREGYTLEYT showed intermediate potency (IC_50_ = 2.64 and 4.24 mM, respectively), while SSMLGLMGGPV was the weakest among the active sequences (IC_50_ = 6.09 mM). Concurrently, experimental ABTS radical scavenging assays determined antioxidant capacities ranging from 1.27 to 5.64 mM Trolox equivalents across the eight sequences **(**Table 2**)**. LGFGADRAGPQ exhibited the highest antioxidant capacity (5.64 mM Trolox equivalents) among all synthesized peptides. The activity likely stems from its aromatic phenylalanine residue positioned favourably for electron donation. The superior experimental activity aligns with mechanistic understanding that aromatic amino acids contribute substantially to antioxidant functionality through electron-donating capacity and radical-stabilizing resonance structures.

These experimental bioassays following chemical synthesis confirm the bioactive potential of the computationally selected sequences and provide direct evidence of their contribution to the overall functionality of fraction F5, warranting further mechanistic investigation and exploration in functional food applications. Comparison with literature values reported on a molar basis places the present sequences within the range documented for collagen-derived ACE-inhibitory peptides. The lead peptide LGLGADMFHR (IC_50_ = 0.52 mM) is comparable in potency to GPPGPPGL from eel (*Anguilla japonica*) bone collagen (IC_50_ = 0.54 mM; Xiang et al., 2024), whereas extensively purified single sequences such as VGLFPSRSF from tilapia skin collagen exhibit stronger inhibition (IC_50_ = 0.061 mM; Dong et al., 2024). The moderate potency observed here is consistent with expectations for sequences identified directly from a complex hydrolysate without iterative affinity-guided purification, and confirms that porcine tracheal cartilage yields ACE-inhibitory peptides of competitive bioactivity among collagen sources. The observed structure-activity relationships established through synthesis and bioassay testing under standardised conditions provide definitive evidence of peptide functionality. Moreover, isoelectric point analysis revealed substantial variation (3.01 to 11.13), indicating diverse electrostatic characteristics across identified sequences. This electrostatic diversity may influence interactions with charged residues within enzyme active sites, potentially affecting binding affinity and inhibitory potency. In silico cell-penetrating peptide prediction yielded scores spanning 0.11 to 0.24, suggesting moderate membrane permeability potential. While these scores fall below thresholds typically associated with highly efficient cell penetration, they indicate that the peptides may possess some capacity for membrane interaction, potentially relevant for intestinal absorption. In silico toxicity prediction algorithms classified all sequences as non-toxic, an essential prerequisite for functional food applications. Additionally, the molecular weight range of identified peptides (870 to 1565 Da) falls within dimensions favourable for oral bioavailability, as peptides below 3000 Da can undergo intestinal absorption through paracellular transport and carrier-mediated mechanisms (Vermeirssen et al., 2002). These eight characterized peptides represent key bioactive components identified within fraction F5 and serve as molecular markers for understanding the hydrolysate's composition and mechanism of action. The experimentally determined bioactivity of these synthesized peptides, combined with their favourable physicochemical properties (appropriate molecular weight, non-toxicity, moderate membrane permeability), confirms their presence as functional sequences in the original hydrolysate. The collective bioactivity of fraction F5 measured in Section 3.5 represents the synergistic contribution of these identified peptides along with other co-existing sequences within the complex mixture. The characterization of these specific sequences provides essential information for patent documentation and product standardization, and establishes a foundation for subsequent studies examining peptide stability, absorption, and bioavailability under physiological conditions.

### 3.7 Molecular docking and binding mechanisms of the most potent peptide inhibitors

**Fig. 5:**
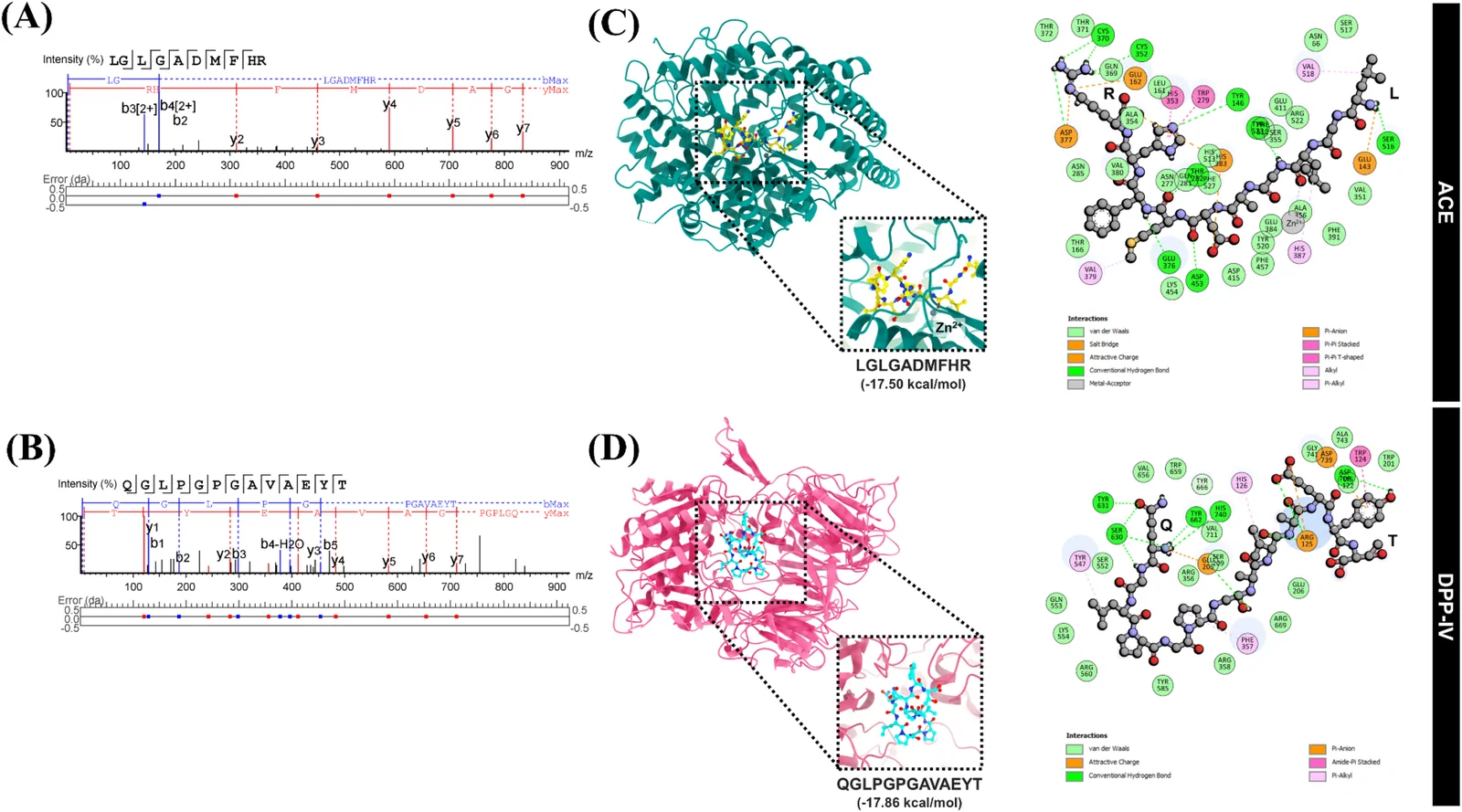
Sequence validation and predicted binding of the two lead peptides. Annotated MS/MS spectra of **(A)** LGLGADMFHR and **(B)** QGLPGPGAVAEYT (b−/y-ion series; PEAKS score > 90%; lower panels, per-fragment mass error). Docking poses of **(C)** LGLGADMFHR in ACE (PDB 4C2P; Zn^2+^ coordination and H-bonds with Cys352, Glu376, Asp453) and **(D)** QGLPGPGAVAEYT in DPP-IV (PDB 2RIP; contacts with Ser630 and His740).

The de novo sequence assignments of the two lead peptides were first verified by their annotated MS/MS spectra, which displayed continuous b- and y-ion series with mass errors within ±0.3 Da, confirming the identities of LGLGADMFHR and QGLPGPGAVAEYT (Fig. 5A and B). These two sequences, exhibiting the most potent experimentally determined bioactivities as LGLGADMFHR for ACE (IC_50_ = 0.52 mM) and QGLPGPGAVAEYT for DPP-IV (IC_50_ = 1.84 mM). Those were then subjected to molecular docking to elucidate their binding mechanisms at atomic resolution (Fig. 5C and D). LGLGADMFHR achieved stable docking positioning within the ACE active site (Fig. 5C). Computational modelling suggests that the most critical interaction would involve metal coordination between the peptide carbonyl group and the catalytic Zn^2+^ ion. This interaction represents the hallmark of competitive ACE inhibition, effectively displacing water molecules necessary for substrate hydrolysis. Metal coordination to the zinc cofactor proves particularly critical for achieving potent ACE inhibition, as this interaction directly interferes with the catalytic mechanism while simultaneously anchoring the inhibitor within the active site pocket (Silva-Velasco et al., 2024).

Modelling predicted formation of multiple conventional hydrogen bonds with active site residues including Cys352, Glu376, and Asp453 (Antony et al., 2024). These hydrogen bonding interactions provide additional stabilization beyond the primary metal coordination, contributing to overall binding affinity through cumulative energetic effects. Attractive charge interactions were predicted through Glu143 and Glu162, residues that line the substrate-binding groove and participate in electrostatic stabilization of bound ligands. Van der Waals contacts emerged in docking poses with residues lining the S1 and S2 subsites, including Val351 and catalytic residues Glu384 and Glu411. Glu384 plays a crucial catalytic role in the ACE mechanism, acting first as general base to activate the nucleophilic water molecule, then as general acid to donate a proton initiating peptide bond hydrolysis. The predicted positioning of LGLGADMFHR in proximity to these catalytic residues suggests a plausible molecular basis for the observed experimental ACE inhibition through occupation of the substrate-binding pocket. Specifically, the hydrophobic C-terminal residues of LGLGADMFHR (methionine, phenylalanine, histidine, arginine) were predicted to establish favourable van der Waals interactions with the hydrophobic pockets lining the ACE active site. This predicted binding mode aligns with established structure-activity relationships for ACE inhibitory peptides, where C-terminal hydrophobic residues frequently contribute substantially to binding affinity through hydrophobic interactions with subsites S1 and S1’ (Natesh et al., 2003). The presence of aspartic acid within the sequence provides potential for metal coordination, while the overall sequence length (10 amino acids, 1115.55 Da) falls within the size range commonly reported as favourable for ACE active site accommodation (2–12 amino acids; <1500 Da; Taghizadeh et al., 2025; W. Hu et al., 2025), allowing penetration into the substrate-binding pocket without excessive steric hindrance.

QGLPGPGAVAEYT modelling suggested occupation of the DPP-IV active site groove **(**Fig. 5D**)**, with predicted formation of extensive interactions with catalytic residues. Computational analysis indicated stabilization primarily through hydrogen bonds involving Ser630 and His740, key catalytic residues in the DPP-IV mechanism. In the DPP-IV catalytic mechanism, the Ser630 hydroxyl group acts as nucleophile, activated by His740 acting as general base. The predicted hydrogen bonding with these residues suggests a potential molecular mechanism underlying the experimentally observed competitive inhibition through occupation of the catalytic site, preventing substrate access and turnover. Arg125, located in the S2 pocket which recognizes the P2 position of substrates, was predicted to participate in both hydrogen bonding and attractive charge interactions, anchoring the peptide backbone within the binding groove. Further, an amide-π stacked interaction with Trp124 emerged prominently in docking poses, suggesting this interaction may contribute substantially to binding within the hydrophobic pocket characteristic of the DPP-IV active site. Trp124 forms part of the S1 pocket, which accommodates hydrophobic residues at the P1 position of substrates and inhibitors. The multiple proline residues within QGLPGPGAVAEYT (positions 4 and 6) provide conformational rigidity through their cyclic structure, potentially pre-organizing the peptide into conformations favourable for DPP-IV recognition. This structural feature aligns with DPP-IV's substrate specificity for proline at the P2 position, where the enzyme preferentially cleaves X-Pro dipeptides from N-termini (Thoma et al., 2003).

The molecular docking analysis offers mechanistic interpretation that rationalises the experimentally determined IC_50_ values, with predicted binding modes consistent with established structure–activity relationships for ACE and DPP-IV inhibitors. While docking results are theoretical models constrained by rigid-protein assumptions, neglect of induced-fit conformational adaptation, and simplified scoring functions, the concordance between predicted binding poses and experimental bioactivities supports the conclusion that tracheal cartilage harbors sequences with structural features favourable for cardiovascular-relevant applications.

### 3.8 Stability and bioactivity transformation during simulated gastrointestinal digestion

**Fig. 6:**
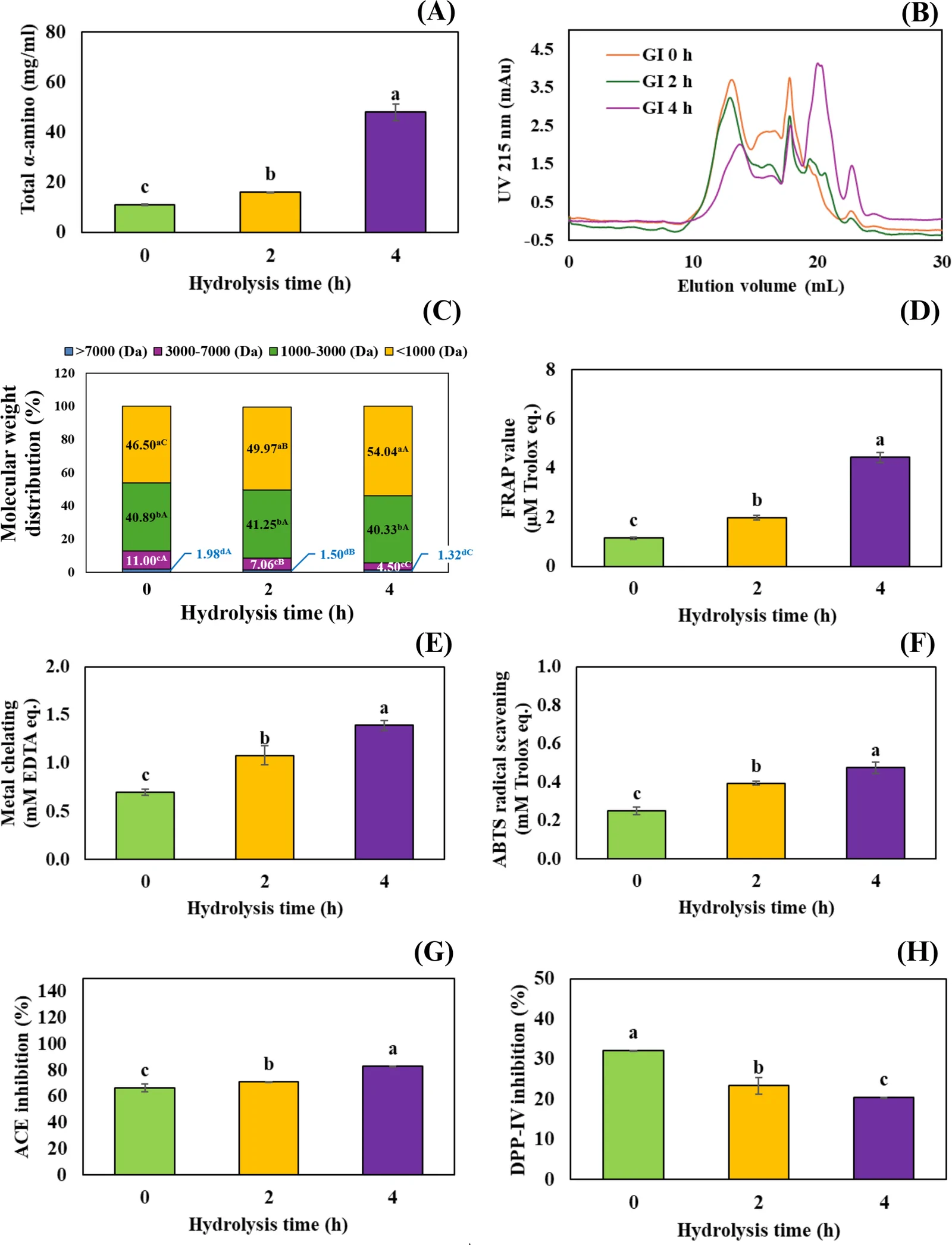
Transformation of peptide characteristics and bioactivities during in vitro gastrointestinal digestion of 4-h Neutrase hydrolysate. Samples were analysed at three digestion phases: before digestion (GI 0 h), after gastric digestion (GI 2 h), and after intestinal digestion (GI 4 h). **(A)** Total α-amino groups (mg/mL). **(B)** Size exclusion chromatography (SEC) profiles showing molecular weight distribution. **(C)** Bar chart showing percentage distribution of peptide molecular weight ranges across digestion time points. **(D)** Ferric-reducing antioxidant power (FRAP). **(E)** Metal chelating activity. **(F)** ABTS radical scavenging activity. **(G)** ACE inhibitory activity. **(H)** DPP-IV inhibitory activity. Different uppercase letters (^A-D^) indicate significant differences across digestion time points (*p* ≤ 0.05). Different lowercase letters (^a-c^) indicate significant differences among digestion time points (*p* ≤ 0.05). Assay sample dilutions relative to stock: SEC, 5×; FRAP, metal-chelating, and ABTS assays, 2.5×; ACE and DPP-IV assays, 8×. Values represent mean ± SD (*n* = 3).

The 4-h Neutrase hydrolysate was subjected to standardised INFOGEST simulated gastrointestinal digestion to assess bioactivity stability under conditions mimicking the human digestive tract **(**Fig. 6**)**. Progressive molecular weight reduction accompanied sequential digestion phases. Total α-amino group content increased significantly throughout digestion **(**Fig. 6A**)**, rising from 11.23 mg/mL before digestion to 17.31 mg/mL after gastric digestion and 47.86 mg/mL after intestinal digestion (*p* ≤ 0.05), indicating extensive peptide bond cleavage by gastric and pancreatic proteases (Musaimi, 2024). Size exclusion chromatography revealed corresponding molecular weight shifts **(**Fig. 6B**)**, with progressive shift of elution peaks toward lower retention volumes, indicating generation of smaller peptides.

Molecular weight distribution analysis **(**Fig. 6C**)** provided quantitative evidence of proteolytic progression. The proportion of peptides <1000 Da increased substantially from 46.50% before digestion to 49.97% after gastric digestion and 54.04% after intestinal digestion. Correspondingly, the 1000–3000 Da fraction showed moderate changes from 40.89% to 41.25% to 40.33%, while the 3000–7000 Da fraction decreased from 11.00% to 7.06% to 4.50%, and the >7000 Da fraction decreased from 1.98% to 1.50% to 1.32%. This progressive shift toward smaller molecular weights demonstrates extensive proteolytic cleavage, generating predominantly di-, tri-, and tetrapeptides (200–1000 Da) suitable for intestinal absorption through paracellular routes and peptide transporter-mediated mechanisms (PepT1).

Critically, bioactivity assessment revealed progressive enhancement rather than degradation across multiple functional properties. Ferric-reducing antioxidant power increased from 1.38 μM Trolox equivalents before digestion to 2.09 μM after gastric digestion and 4.38 μM after intestinal digestion (p ≤ 0.05, Fig. 6D), representing 217% enhancement. This elevation suggests that gastrointestinal proteolysis liberated amino acid residues with electron-donating capacity, potentially including aromatic and sulfur-containing residues. Metal chelating capacity displayed a progressive, two-step increase, rising from 0.69 mM EDTA equivalents to 1.06 mM during gastric digestion, then further increasing to 1.37 mM after intestinal digestion (p ≤ 0.05, Fig. 6E), representing 99% overall enhancement. ABTS radical scavenging activity increased from 0.24 mM Trolox equivalents before digestion to 0.39 mM after gastric digestion and 0.47 mM after intestinal digestion (p ≤ 0.05, Fig. 6F), representing 96% enhancement.

Most significantly for cardiovascular relevance, ACE inhibition demonstrated substantial progressive enhancement **(**Fig. 6G**)**. Inhibitory activity increased from 73.5% before digestion to 84.6% after gastric digestion and 91.3% after intestinal digestion, representing 24% relative enhancement (p ≤ 0.05). This progressive strengthening throughout simulated gastrointestinal transit represents the most functionally relevant observation, as ACE inhibition directly correlates with blood pressure regulation potential. The enhancement pattern suggests that gastrointestinal proteolysis liberates peptide sequences with superior ACE binding affinity compared to the starting material. In contrast, DPP-IV inhibition declined from 31.24% before digestion to 23.16% after gastric digestion and 20.37% after intestinal digestion (p ≤ 0.05, Fig. 6H), representing 35% reduction and indicating susceptibility to proteolytic degradation. This progressive enhancement of ACE inhibition through digestion contrasts with the frequent observation that bioactive peptides lose activity upon digestive proteolysis (Zhou et al., 2025), aligning instead with reports of digestion-induced activation of food-derived ACE-inhibitory hydrolysates (Chen et al., 2025). The divergent fate of ACE inhibition (enhanced) versus DPP-IV inhibition (diminished) under identical conditions is consistent with target-specific effects of gastrointestinal proteolysis on multifunctional hydrolysates (Sangsawad et al., 2017), reinforcing that digestive stability must be assessed per bioactivity rather than assumed uniform.

The observed bioactivity enhancement may stem from several plausible mechanisms, including resistance of bioactive peptides to proteolytic degradation, concentrating overall activity as less active components undergo removal; cryptic bioactivity liberation, where gastrointestinal proteases cleave larger parent peptides into smaller, more potent fragments (Chen et al., 2025); or combined mechanisms operating simultaneously. In silico cleavage site analysis **(**Table 2**)** suggests potential fragmentation during digestion. However, proline-containing sequences sometimes resist proteolysis despite harboring theoretical cleavage sites, due to conformational constraints. Definitive determination of peptide fate would require direct peptidomic tracking through digestion phases, which represents an important avenue for future investigation.

Regardless of precise mechanism, the practical translational implication is clear. The peptide mixture maintains and enhances cardiovascular-relevant bioactivities throughout simulated gastrointestinal digestion rather than undergoing functional degradation. These findings establish that tracheal cartilage peptide hydrolysates maintain functionality through gastrointestinal transit, positioning them as promising candidates for oral delivery in functional food applications. Future studies employing in vivo absorption tracking, peptidomic identification of absorbed fragments, and clinical validation in spontaneously hypertensive rat models would constitute essential next steps to establish whether oral administration reduces blood pressure and ultimately determine translational potential for cardiovascular health applications.

## 4 Conclusions

This investigation establishes porcine tracheal cartilage as a viable substrate for generating multifunctional bioactive peptides through enzymatic hydrolysis, addressing critical gaps in collagen by-product valorisation. Comparative protease evaluation demonstrated that Neutrase achieved superior ACE inhibitory activity (73.5% for the 4-h hydrolysate selected for downstream analysis) despite lower degree of hydrolysis than Papain, challenging simplistic assumptions that equate extensive proteolysis with enhanced bioactivity and revealing activity-dependent structure-function relationships. Systematic molecular weight fractionation confirmed that peptides below 3 kDa exhibited optimal cardiovascular bioactivities, with reversed-phase chromatography demonstrating that high hydrophobicity further enhanced ACE inhibition. LC-MS/MS identified eight bioactive sequences (870–1565 Da) from Type II collagen; chemical synthesis and experimental assays confirmed LGLGADMFHR as the most potent ACE inhibitor (IC_50_ = 0.52 mM), QGLPGPGAVAEYT as the lead DPP-IV inhibitor (IC_50_ = 1.84 mM), and LGFGADRAGPQ as the strongest antioxidant (5.64 mM Trolox equivalents). Molecular docking elucidated binding mechanisms involving zinc coordination and extensive hydrogen bonding with catalytic residues, providing molecular-level insights into structure-activity relationships. Most significantly, standardised INFOGEST simulated gastrointestinal digestion enhanced ACE inhibition to 91.3% and progressively increased antioxidant capacity, demonstrating cryptic bioactivity liberation rarely documented for collagen-derived peptides. While bulk bioactivity measurements preclude definitive mechanistic determination and in vivo efficacy studies remain essential for clinical translation, these findings position tracheal peptides as promising clean-label ingredients for functional foods targeting hypertension and oxidative stress. Collectively, this investigation demonstrates the translational value of integrating comparative enzyme evaluation, size-dependent fractionation, sequence identification, and gastrointestinal stability assessment in sustainable meat processing waste valorisation.

## Ethical Statement - Studies in humans and animals

This research did not include any studies involving human participants or animal subjects. Therefore, ethical approval for human or animal testing was not required for this work.

## Declaration of generative AI use

During the preparation of this work, the authors used Claude (Anthropic) to improve the grammar and language of the manuscript. After using this tool, the authors reviewed and edited the content as needed and take full responsibility for the content of the publication. The graphical abstract was created using BioRender (license: TA296RAW2Y).

## CRediT authorship contribution statement

**Rayudika Aprilia Patindra Purba:** Writing – review & editing, Writing – original draft, Validation, Conceptualization. **Suchanya Sinsranoi:** Writing – original draft, Visualization, Validation, Investigation, Formal analysis. **Phanthipha Laosam:** Writing – review & editing, Validation, Methodology. **Phornpilat Senanok:** Validation, Formal analysis. **Pichitpon Luasiri:** Writing – original draft, Validation, Formal analysis. **Pornphutthachat Sota:** Writing – review & editing, Validation. **Nattapol Pongsamai:** Writing – review & editing, Validation. **Saranya Suwanangul:** Writing – review & editing, Validation. **Jukkrapong Pinyo:** Writing – review & editing, Validation, Methodology. **Sasikan Katemala:** Writing – review & editing, Validation, Formal analysis. **Saruttiwong Boonkong:** Visualization, Formal analysis. **Kamonpan Sanachai:** Writing – review & editing, Visualization, Validation, Formal analysis. **Papungkorn Sangsawad:** Writing – review & editing, Supervision, Methodology, Investigation, Funding acquisition, Formal analysis, Conceptualization.

## Declaration of competing interest

The authors declare that they have no known competing financial interests or personal relationships that could have appeared to influence the work reported in this paper.
